# Supplementary material for: Innovative tracking, active search and follow-up strategies for new leprosy cases in the female prison population
Source: PLoS Negl Trop Dis. 2021 Aug 20;15(8):e0009716. doi: 10.1371/journal.pntd.0009716 (PMC8409633; doi:10.1371/journal.pntd.0009716)
Supplement: Attachment — Submitted filename: Author Responses Jul 23, 2021.docx [file pntd.0009716.s001.docx]

July 21, 2021.

Carlos Franco-Paredes

Associate Editor

PLOS Neglected Tropical Diseases

Richard Phillips

Deputy Editor

PLOS Neglected Tropical Diseases

Sub: PNTD-D-21-00620

Dear Editors,

We would like to thank you for all comments about our manuscript: “Innovative tracking, active search and follow-up strategies for new leprosy cases in the female prison population” because they appointed some important aspects to improve it.

The requested edits were considered and they are in a red in the manuscript.

**Remarks**

**Reviewer's Responses to Questions**

**Methods**

-Are the objectives of the study clearly articulated with a clear testable hypothesis stated?

-Is the study design appropriate to address the stated objectives?

-Is the population clearly described and appropriate for the hypothesis being tested?

-Is the sample size sufficient to ensure adequate power to address the hypothesis being tested?

-Were correct statistical analysis used to support conclusions?

-Are there concerns about ethical or regulatory requirements being met?

Reviewer #1: The objectives of the study are clear. But what they want to proof you can doubt. Population is well descibed and appropiate to test the hypothesis. But they try to do too much.

Author’s comments: Really, this manuscript brought lots of results, because in the project MHBrasil, we proposed to use many tools to improve the leprosy diagnosis. Thanks for your comments.

Reviewer #2:

-Are the objectives of the study clearly articulated with a clear testable hypothesis stated? – Yes

-Is the study design appropriate to address the stated objectives? - Yes

-Is the population clearly described and appropriate for the hypothesis being tested? – Yes

-Is the sample size sufficient to ensure adequate power to address the hypothesis being tested? – Yes

-Were correct statistical analysis used to support conclusions? – Yes

-Are there concerns about ethical or regulatory requirements being met? - No.

Author’s comments: Ok.

**Results**

-Does the analysis presented match the analysis plan?

-Are the results clearly and completely presented?

-Are the figures (Tables, Images) of sufficient quality for clarity?

Reviewer #1: Yes

Author’s comments: Ok.

Reviewer #2: The analysis, results and tables/figures are well presented

Author’s comments: Ok.

**Conclusions**

-Are the conclusions supported by the data presented?

-Are the limitations of analysis clearly described?

-Do the authors discuss how these data can be helpful to advance our understanding of the topic under study?

-Is public health relevance addressed?

Reviewer #1: The do that well.

Author’s comments: Ok.

Reviewer #2: Authors should check the Summary and General Comments.

Author’s comments: Ok.

**Editorial and Data Presentation Modifications?**

Use this section for editorial suggestions as well as relatively minor modifications of existing data that would enhance clarity. If the only modifications needed are minor and/or editorial, you may wish to recommend “Minor Revision” or “Accept”.

Reviewer #1: Rewrite.

Author’s comments: Ok.

Reviewer #2: There is a need for spelling corrections

Author’s comments: Ok.

**Summary and General Comments**

Use this section to provide overall comments, discuss strengths/weaknesses of the study, novelty, significance, general execution and scholarship. You may also include additional comments for the author, including concerns about dual publication, research ethics, or publication ethics. If requesting major revision, please articulate the new experiments that are needed.

Reviewer #1: Dear Authors,

Having read your paper on using your questionnaire in town and in a male prison, I now see it applied in a female prison. What is new? Are there differences is it a repetition or something new?

Author’s comments: In this paper, we just consolidated the results with LSQ in a female population living in a different penitentiary regime (close regime). One other important finding with LSQ in this population was to find that when more than one question was marked in the LSQ means a 12.8-fold higher to have the disease than a subject who marked only one or none. Finally, the work brought one prospective analyzing of the individuals during 36 months applying the LSQ and anti-PGL-I for three times, consolidating both as significant tools for leprosy diagnosis and also for the follow up of contacts.

It is a beautiful report of a careful done survey. But more a thesis then a paper. As clinician I enjoy reading it. But hardly anything new. Only it made me more critical on the LSQ. I wonder whether except for testing this test, a prison is the right place were it is needed. What is new?

Author’s comments: Sure, we have had many results, but the most important was to use the LSQ and APG-I for prospective analyzing as described above.

Just a few comments going through the paper:

I would have the English checked.

Author’s comments: Ok, we have done it.

The paper certainly is of local interest and findings such a great number of patients with leprosy shows that the study was worth doing and makes you wonder what of the real number of leprosy cases is in underprivileged areas.

Author’s comments: Ok.

You mention that 3 patients were diagnosed 18 and 36 months later. Why were they missed before? What extra signs did develop? This is something you may learn from.

Author’s comments: All patients were diagnosed with leprosy according to the Brazilian Ministry of Health guidelines considering at least one of the following signs/symptoms: a) lesion(s) and/or area(s) of the skin with changes in thermal and/or painful and/or tactile sensitivity; b) thickening of the peripheral nerve(s), associated with sensory and/or motor and/or autonomic changes; and/or c) presence of *M. leprae* confirmed by intradermal smear microscopy or skin biopsy [12]. However, in active search actions in the community, there are cases in which these items are not always obvious on clinical examination. Therefore, we use the tools to define areas that are more susceptible to neural and/or dermatological damage prone to leprosy using the LSQ, as well as ultrasound of peripheral nerves to define nerve hypertrophy, as well as more sensitive tests for bacillary detection such as PCR and/or the bacillus-specific immune response by Anti-PGL-I.

About these 3 additional cases, they had only marked questions in LSQ, and nothing more in the first and second visits. In the third visit, the patients presented more symptoms, alterations during the peripheral nerve exam, and the physical examination by monofilaments presented altered points on feet, or the annti-PGL1 increased. So, we decided to improve the investigation with ultrasound to prove the enlargement of the nerves (one of the cardinal criteria) and also the eletroneuromiography to detect the neural impairment.

Even in healthy persons the lower limb has lower values then the upper limbs. What was the criteria to call them affected?

Author’s comments: Thanks for your comment. We added these information about alterations on the hands (does not fell 0.05g-force) and feet (does not fell 0.2g-force) according to SW monofilament tactile test.

Lines 212-218: “Only the hands and feet tactile sensitivities seems to have relation with the anti-PGL-I positivity highlighting the Semmes-Weinstein monofilaments (SMW) used to detect these neuropathic signs for the diagnosis of leprosy. Curiously, only 7 (35%) of LG patients presented some alterations (does not fell 0.05g-force) in the hands detected by SMW, while 14 (70%) of them presented sensitivity alterations (does not fell 0.2g-force) in the feet (p=0.003).”

You evaluated all 404 inmates. Was there an incentive?

Author’s comments: Yes, we evaluated everyone with no incentive for anyone.

59 you did not revaluate the patients but the inmates. These 6 were found after how long time? Are these different from the 3 after 18 and 36 months?

Author’s comments: Sure, you are right. We changed the abstract for “Afterwards, we reevaluated the inmates a 6 additional leprosy cases were diagnosed….”

Were the 6 not suspected after LSQ. How many would be discovered when only the positive LSQ persons were evaluated. Why is it more efficient with the LSQ when you evaluate all inmates?

Author’s comments: The application of LSQ is a new tool and we have applied it for all inmates. Different from male population, in this female population all new cases were LSQ+.

The port of exit of the bacilli is most likely upper respiratory tract, but port of entre may be different.

Author’s comments: Ok.

76 over crowding and precarious condition (what does that imply?) Maybe you mean what is written in the next sentence.

Author’s comments: Yes, the next sentence clarified your doubt.

All got the questionnaire, and all were examined. What was the contribution of the LSQ? Except assessing the questionnaire again.

Author’s comments: The LSQ improved and spread the knowledge about leprosy in the penitentiary, besides the using it according to the answers received to think about leprosy by the health professionals. As we know the benefits of LSQ, it has initially a function of alarm to health care to track the individuals.

The nerve ultrasonography was a separate research or did it contribute to the diagnosis. Was there any patient diagnosed that was missed otherwise?

Author’s comments: Yes, we have also another student working specifically with nerve ultrasound in the field, but in this case, considering the high level of APGL-I positive inmates in the penitentiary, we decided to add the nerve ultrasonography mainly in the cases with high serology and many positive questions to get to fill one of the cardinal criteria for the diagnosis as the nerve enlargement taking into account that some patients had no lesion or area of evident sensitivity change, as described above.

Same question for anti-PGL1. And in a way also for the DNA extraction from the smear.

Did you compare between microscopy and Staining for AFB?

Author’s comments: Well, as described for LSQ, only this positive result did not represent the diagnosis of leprosy. We followed the patients during the time and according to the increase of clinical signs and symptoms or the increase of the ELISA anti-PGL1 index we decided to do other complementary exams as US and eletroneuromiography. No, we did not do microscopy analysis considering the low sensitivity of the bacilloscopy analysis for these kinds of cases.

You say the sensory testing was done at start and 6 month and at the end. I may hope also when needed. In table 1 check the lines in the last 2 columns. How large do you expect is the chance of having leprosy with a positive LSQ compared with a positive anti PGL-1? You first paper by using it in town give me a good feeling. With the male prisoners I still thought it a contribution. But now I start to doubt. Statistics leave out individuals. Because like positive or negative anti PGL-1 you only have an indication. And then anti-PGL-1 can be used for epidemiology and LSQ not.

Author’s comments: As described above, in active search actions in the community, there are cases in which the cardinal signs for leprosy diagnosis are not always obvious on clinical examination. Therefore, we use the tools to define areas that are more susceptible to neural and/or dermatological damage prone to leprosy using the LSQ, as well as ultrasound of peripheral nerves to define nerve hypertrophy, as well as more sensitive tests for bacillary detection such as PCR and/or the bacillus-specific immune response by Anti-PGL-I. We propose to use all tools together to empower the health professionals mainly to detect the mild leprosy cases avoiding disabilities and breaking the bacillary transmission.

218 The longer the peripheral nerves the more chance you have to find abnormalities: leprosy is not different from diabetes. PGL-1 by itself has been shown to be able to damage the myeline sheath.

Author’s comments: Sorry, but I did not understand your sentence very well, but we will try to add some comments. About neuropathy in both diseases, in diabetes we did not have the enlargement of peripheral nerves like in leprosy, which we can find asymmetry and focality very characteristically. Also, about hands tactile sensitivity, in diabetes we can find one symmetrical distribution of the alterations mainly in the feet bilaterally while in leprosy the alteration also shows us the asymmetry and focal pattern of neuropathy. The correlation between sensitivity test with PGL-I significantly improves the specificity for leprosy diagnosis and may be about the involvement of *M. leprae* in the damage of myeline sheath.

On 243 you mention that the patients were measured monthly, as it should with esthesiometry evaluations. Before you mentioned 6 and 12 months. The discussion is very long this is mainly because you studied so many parameters. The “thesis” could be separated in smaller papers and published in appropriate journals.

Author’s comments: We agree with you about the size of the manuscript, but we really have used many parameters to consolidate our diagnosis among the inmates, one so discriminated population. On the other hand, we also proposed to do one clinical follow up for 3 years becoming difficult put many data in a short manuscript without run the risk to change our main goal to show the hidden endemic of leprosy in a female penitentiary.

Reviewer #2: The results of this work are very interesting and can contribute to the early diagnosis of leprosy. Some aspects caught the attention of this reviewer, highlighted below:

• In the abstract: “The NCDR in this population showed hidden leprosy like among males in prison, as well as the efficacy of a search action on the part of a specialized team with the aid of the LSQ as an auxiliary tracking tool”.

The author compares the results obtained from this study with a similar study carried out by the research team in the imprisoned male population. However, this consideration in the abstract is inappropriate. In the discussion of the results, this aspect would be relevant.

Author’s comments: Thanks for your comment and we agree with you. So, we changed the interpretation of the abstract for:

Lines 44-46: “The NCDR in the female prison population showed hidden endemic of leprosy , as well as the efficacy of a search action on the part of a specialized team with the aid of the LSQ and anti-PGL-I serology as an auxiliary tracking tools.”

• In the discussion:

Lines 371-374: “This fact may be related to the quality of the health team involved in the action since all the specialized professionals of the present center (National Reference Center of Sanitary Dermatology – Leprosy, Clinical Hospital of Ribeirão Preto Medical School at, University of São Paulo) are more attuned and trained to recognize a diagnosis of leprosy in its more subtle manifestations”.

Despite the different epidemiological scenarios between the two regions, (São Paulo and Recife), it must be considered that the methodology used for the two studies are different and that no value judgment is appropriate in relation to the clinical expertise of the authors involved in the study cited as a reference.

Author’s comments: Thanks a lot for your comment!

We rewrote the sentence between lines 372-378 for:

“This fact may be related to the health team involved in the action since all the specialized professionals of the present center (National Reference Center of Sanitary Dermatology – Leprosy, Clinical Hospital of Ribeirão Preto Medical School at, University of São Paulo) are more attuned and trained to recognize a diagnosis of leprosy in its more subtle manifestations besides the additional tools used as LSQ, anti-PGL-I serology.”

Furthermore, considering leprosy a "hidden endemic" in a country with continental dimensions such as Brazil, it is possible to analyse these results from another aspect, such as the lack of expertise of professionals in the region where this same study was carried out.

Author’s comments: Yes, sure. This is one important point of view.

The well-trained team of this work, under revision using a different and more accurate methodology, arrived at results close to this same group mentioned, which used the most common and routinely available tools in the field [1].

Author’s comments: Yes, we changed the context in the manuscript. But we should consider the difference between regions where in Recife the detection rate were 3 times more than in Ribeirão Preto. The reference [1] was cited in the manuscript as reference [14].

Lines 383-386: “The high proportion of multibacillary patients observed in our study should be considered as an important flare gun of transmissibility of the disease to health services in the strategies of active search and consequently of breaking the chain of transmission, since the multibacillary form, presents in 95 % of the diagnosed cases, is recognized by its great power of transmission”. (There are spelling that needs correction).

Author’s comments: Thanks a lot. We changed completely this paragraph (lines 387-394).

It is possible that this conclusion was reinforced if there were parameters for bacilloscopic evaluation, which is a gap in this study, also in relation to PCR’s positivity. (The authors’ statement in line 323 is that “There were no new cases of PCR positivity”) Nevertheless, it is true that early diagnosis occurred in the studied groups, using esthesiometry and anti-PGL-I serology.

Author’s comments: We changed the paragraph (lines 387-394). We understood your comments, but considering all parameters used to evaluate the patients became difficult to characterize anyone as paucibacillary.

Lines 387-394: Considering the complex and probably multifactorial transmission of *M. leprae* and *M. lepromatosis* (Ploemacher et al (2020), although no case has been positive on the PCR test in our study, the high proportion of esthesiometry alterations, positive anti-PGL-I serology and multibacillary classification of our patients should be considered as an important *flare gun* of transmissibility of the disease to health services. Such manifestations and multibacillary classification were found similarly in the male prison population according to data which had already been published by our group [11].

However, the transmission of leprosy among cases classified as multibacillary so far is described as being possible due to the elimination of bacilli through the known routes of this group of patients to the outside world. Although leprosy, according to the most recent WHO guidelines, can be classified as multibacillary with the detection of only one compromised nerve, the assertion that these patients listed is this study (at this stage of the disease) are responsible for maintaining the epidemiological chain of transmission can be hasty, for the time. The authors seem to understand this aspect, in lines 409-411. The leprosy transmission is complex is multifactorial.

Author’s comments: Yes, you are right.

According Ploemacher and cols (2020): “This entails integrating human, animal, and environmental health aspects to further elucidate the transmission mechanisms and patterns of *M. leprae* and *M. lepromatosis*. In addition, geographically tailored methods–combining epidemiological, laboratory, and anthropologic data–are needed to better understand the ecological differences between leprosy pockets” [2].

Author’s comments: We have added this reference in the paper.

Once they realized, the authors need to clarify or discuss the lack of positivity in the PCR test.

Author’s comments: Thanks. We added in the manuscript:

Lines 393-396: “Regarding the lack of positivity in the conventional PCR test for slit skin smears, it may be related to the diagnosis of mild leprosy in our cases that, despite having multiple sites of nervous involvement, these cases seem to have a low bacillary load.”

Lines 449-451: “We believe that the greatest focus on the ulnar nerve in these studies may be due to the fact this nerve is located in an area that is more accessible to traumas in terms of daily movements and activities, in contrast to the fibular and tibial nerves”. This statement deserves attention, considering that the plantar regions whose innervation is the responsibility of the posterior tibial nerve are as susceptible (or perhaps more susceptible) to trauma than those innervated by the ulnar nerve. There are several studies that offer attention to the posterior tibial nerve in leprosy [3,4,5,6].

Author’s comments: Thanks for your comment, but in this point we mentioned about the anatomical exposure of ulnar nerve and not about the innervation area. Because of this point of view, we changed the paragraph as described below and we added two references about tibial nerve.

Lines 467-472: In this respect, we believe that the greatest focus on the ulnar nerve in these studies may be due to the fact this nerve is located between medial epicondyle of humerus and olecranon, in an superficial area which is more accessible to traumas in terms of daily movements and activities, in contrast to the fibular nerve, located behind of fibular head, and tibial nerve, located posterior to medial malleolus. Another important factor is that the posterior tibial nerve is not easily nor routinely evaluated in electroneuromyography and there is a few numbers of papers about its impairment in leprosy [XXXXXXX].

This work is relevant and looks at a neglected disease in a neglected population. It demonstrates that simple tools such as an esthesiometer and a well-applied questionnaire can significantly contribute to the diagnosis of leprosy. To corroborate their results, however, the authors use sophisticated tools that are unavailable to the vast majority, needing to discuss this important aspect.

Author’s comments: Thanks a lot!

References:

1. Bernardes F Filho, Paula NA, Leite MN, et al. Evidence of hidden leprosy in a supposedly low endemic area of Brazil. Mem Inst Oswaldo Cruz. 2017;112(12):822-828. doi:10.1590/0074-02760170173.

2. Ploemacher T, Faber WR, Menke H, Rutten V, Pieters T. Reservoirs and transmission routes of leprosy; A systematic review. PLoS Negl Trop Dis. 2020;14(4):e0008276. Published 2020 Apr 27. doi:10.1371/journal.pntd.0008276.

3. Gupta BK, Kochar DK. Study of nerve conduction velocity, somatosensory-evoked potential and late responses (H-reflex and F-wave) of posterior tibial nerve in leprosy. Int J Lepr Other Mycobact Dis. 1994;62(4):586-593.

4. Richard B, Khatri B, Knolle E, Lucas S, Turkof E. Leprosy affects the tibial nerves diffusely from the middle of the thigh to the sole of the foot, including skip lesions. Plast Reconstr Surg. 2001;107(7):1717-1724. doi:10.1097/00006534-200106000-00012.

5. Jain S, Visser LH, Praveen TL, et al. High-resolution sonography: a new technique to detect nerve damage in leprosy. PLoS Negl Trop Dis. 2009;3(8):e498. Published 2009 Aug 11. doi:10.1371/journal.pntd.0000498.

6. Wagenaar I, Post E, Brandsma W, et al. Early detection of neuropathy in leprosy: a comparison of five tests for field settings. Infect Dis Poverty. 2017;6(1):115. Published 2017 Sep 1. doi:10.1186/s40249-017-0330-2.

Thank you for your consideration. I look forward to hearing from you.

Sincerely,
